# Serum Uric Acid Levels in Patients with Alzheimer's Disease: A Meta-Analysis

**DOI:** 10.1371/journal.pone.0094084

**Published:** 2014-04-08

**Authors:** Xueping Chen, Xiaoyan Guo, Rui Huang, Yongping Chen, Zhenzhen Zheng, Huifang Shang

**Affiliations:** Department of Neurology, West China Hospital, Sichuan University, Chengdu Sichuan, China; Chiba University Center for Forensic Mental Health, Japan

## Abstract

**Background:**

Serum uric acid (UA) could exert neuro-protective effects against Alzheimer's disease (AD) via its antioxidant capacities. Many studies investigated serum UA levels in AD patients, but to date, results from these observational studies are conflicting.

**Methods:**

We conducted a meta-analysis to compare serum UA levels between AD patients and healthy controls by the random-effects model. Studies were identified by searching PubMed, ISI Web of Science, EMBASE, and the Cochrane library databases from 1966 through July 2013 using the Medical Subject Headings and keywords without restriction in languages. Only case-control studies were included if they had data on serum UA levels in AD patients and healthy controls. Begg's funnel plot and Egger's regression test were applied to assess the potential publication bias. Sensitivity analyses and meta-regression were conducted to explore possible explanations for heterogeneity.

**Results:**

A total of 11 studies met the inclusion criteria including 2708 participants were abstracted. Serum UA levels were not significantly different in AD patients compared to healthy controls (standardized mean difference (SMD) = −0.50; 95% confidence interval (CI): −1.23 to 0.22). Little evidence of publication bias was observed. Sensitivity analyses showed that the combined SMD was consistent every time omitting any one study, except only one study which greatly influenced the overall results. Meta-regression showed that year of publication, race, sample size, and mean age were not significant sources of heterogeneity.

**Conclusion:**

Our meta-analysis of case-control studies suggests that serum UA levels do not differ significantly in AD patients, but there may be a trend toward decreased UA in AD after an appropriate interpretation. More well-designed investigations are needed to demonstrate the potential change of serum UA levels in AD patients.

## Introduction

Alzheimer's disease (AD) is the most common neurodegenerative dysfunction of the central nervous system, characterized by brain atrophy and accumulation of amyloid-plaques and neurofibrillary tangles throughout the cortex by the end of the disease [1]. The clinical symptoms of AD include progressive short-term memory loss, impaired linguistic function, alternations in neuroendocrine functions, emotional dysfunction, declining cognition, and dementia [2]. By 2050 the world will have about 100 million people suffering from AD [3]. Many studies support the theory that oxidative stress and impaired energy metabolism play an important role in the pathogenesis of AD [4], [5], [6], [7], [8], [9], [10], [11], [12]. Cerebral tissue seems to be particularly vulnerable to oxidative damage, because of its high oxygen consumption, low content of antioxidants and high content of polyunsaturated fatty acids of neuronal membranes [13], [14], [15]. The β-amyloid protein itself and other lesion-associated proteins have been reported to cause oxidative damage to neurons [16]. Mitochondrial dysfunction in AD could cause an increased generation of free radicals, and damage the major cell components, including DNA, protein and lipid [17], [18], [19], [20], [21], [22], [23]. Isoprostanes, as a marker of lipid peroxidation, were increased in the brain of AD animal models [24]. Elevated isoprostanes in the brain and cerebrospinal fluid (CSF) of AD patients could be used to monitor the disease progression and the response to therapeutic [25], [26]. In addition, high levels of isoprostanes were also found in plasma and urine, suggesting a possible relationship between central and peripheral markers of oxidative damage [27], [28]. Furthermore, peripheral antioxidants, including uric acid (UA), bilirubin and albumin are strong free radical scavengers. UA is an endogenously produced water-soluble antioxidant [29], [30], accounting for up to 60% of the free radical scavenging capacity in the peripheral system by quenching superoxide and singlet oxygen [31], [32]. These qualities make UA an attractive CNS antioxidant, and its potential protective effects on AD have been reported in prior studies. Some studies suggested that UA levels tend to be low in AD [33], [34], [35], but another report found no difference of UA concentrations in Parkinson's disease (PD), AD and healthy controls [36]. In addition, some studies suggest that patients with higher serum UA levels have a markedly lower risk of progressing from impaired cognitive function to dementia [37], [38], while some studies found that even mild elevation of UA might enhance the risk of cognitive decline in older adults [39]. Other study revealed that patients with normal cognitive function have higher serum UA levels than AD patients [40]. Considering these inconsistent possibilities, we tried to establish what consensus could be reached from various studies by examining the change of serum UA levels in AD patients compared to healthy controls with meta-analytic techniques.

## Methods

### Search strategy

Our systematic review was conducted according to the Meta analysis of Observational Studies in epidemiology (MOOSe) guidelines [41] and the Preferred Reporting Items for Systematic Reviews and Meta-analyses (PRISMA) [42] (Checklist S1). We searched four major electronic databases, including PubMed, ISI Web of Science, EMBASE, and the Cochrane library from 1966 until July 2013 for studies that reported the change of serum UA level in AD patients. No language restrictions and the medical subject heading (MeSH) terms for “Alzheimer's disease” and “urate” or “uric acid” were included in the search strategy. All the articles were retrieved and potentially relevant articles were searched to fulfill the reference lists. Additionally, we also tried to find unpublished datasets, but no studies were suitable for inclusion. The final search was carried out on July 10, 2013.

### Eligibility criteria

All the articles were carefully read and evaluated. Studies were included if they fulfill the following criteria: 1) from case-control studies which compared the serum UA levels between AD patients and healthy controls; 2) defined UA measured at laboratories as exposure factor; 3) clearly stated AD diagnostic criteria; 4) met at least six points of Newcastle-Ottawa Scale (NOS) criteria, which is used for assessing the quality of nonrandomized studies in meta-analysis. If the same or similar population was used in more than one studies, we selected the one with the most complete and relevant data for analysis.

### Exclusion criteria, Data extraction and Quality assessment

Review articles, editorials, commentaries, hypothesis papers, letters that reported no new data, meta-analyses, and abstracts were excluded. The exclusion criteria for this study were clarified as follow: 1) reported on treatment and management of AD; 2) reported associations with dementia occurring before onset of AD; 3) included cases of dementia with Lewy bodies; 4) did not set a control group or provide adequate details on the control group; 5) If control groups >1, the control group most representative of the healthy general population was used; 6) reported on a disease other than AD. Two authors independently evaluated the eligibility of all retrieved studies and extracted the relevant data using a unified form. Finally, discrepancies were discussed until an agreement was reached. A quality score was calculated to assess the quality of the studies according to the NOS criteria. Three major components were collected: selection of the study groups (0–4 points), ascertainment for the exposure of interest in the studies (0–3 points) and quality of the adjustment for confounding (0–2 points). A higher score represents better quality in methodology.

### Data analysis

From each primary study we extracted the data on the study characteristics, including first author's last name, year of publication, country where the study was performed, sample size, mean age, proportion of men, serum UA levels and adjusted covariates. UA values were expressed in International System of Units (umoles/liter). We therefore performed the conversion, from μmol/L to the conventional units (mg/dl), using a rate of 16.81 (1 mg/dl = 59.48 μmol/L).

We examined the presence of heterogeneity across studies using the I^2^ statistic to quantify the percentage of variability that can be attributed to the between-study differences. Heterogeneity among studies was also evaluated using the Chi-square test based on Cochran's Q statistic. If the P-value for heterogeneity is less than 0.10 or I^2^ statistic is greater than 50%, the homogeneous of the studies is rejected. When substantial heterogeneity was detected, the DerSimonian and Laird random effects model was used to compare the change of serum UA levels in AD patients with healthy controls across the studies. The DerSimonian and Laird technique was considered an appropriate pooling method due to the relative heterogeneity generated from the source population in each study. This method weighs individual studies by sample size and variance and generates a pooled point estimate and a 95% confidence interval (95% CI). Sensitivity analyses were conducted to assess the influence of individual result on the pooled estimate. Meta-regression was conducted to explore possible explanation for heterogeneity, especially the effect of four study-level characteristics (year of publication, race, sample size, and mean age) on the serum UA levels in AD. We performed the Egger's test and the Begg's test to assess the potential publication bias, and constructed funnel plots to visualize a possible asymmetry. Two-tailed *P* values less than 0.05 were considered statistically significant. All data analyses were done using Stata 10 (Stata Corp, College Station, TX).

## Results

### Literature search

**Figure 1:**
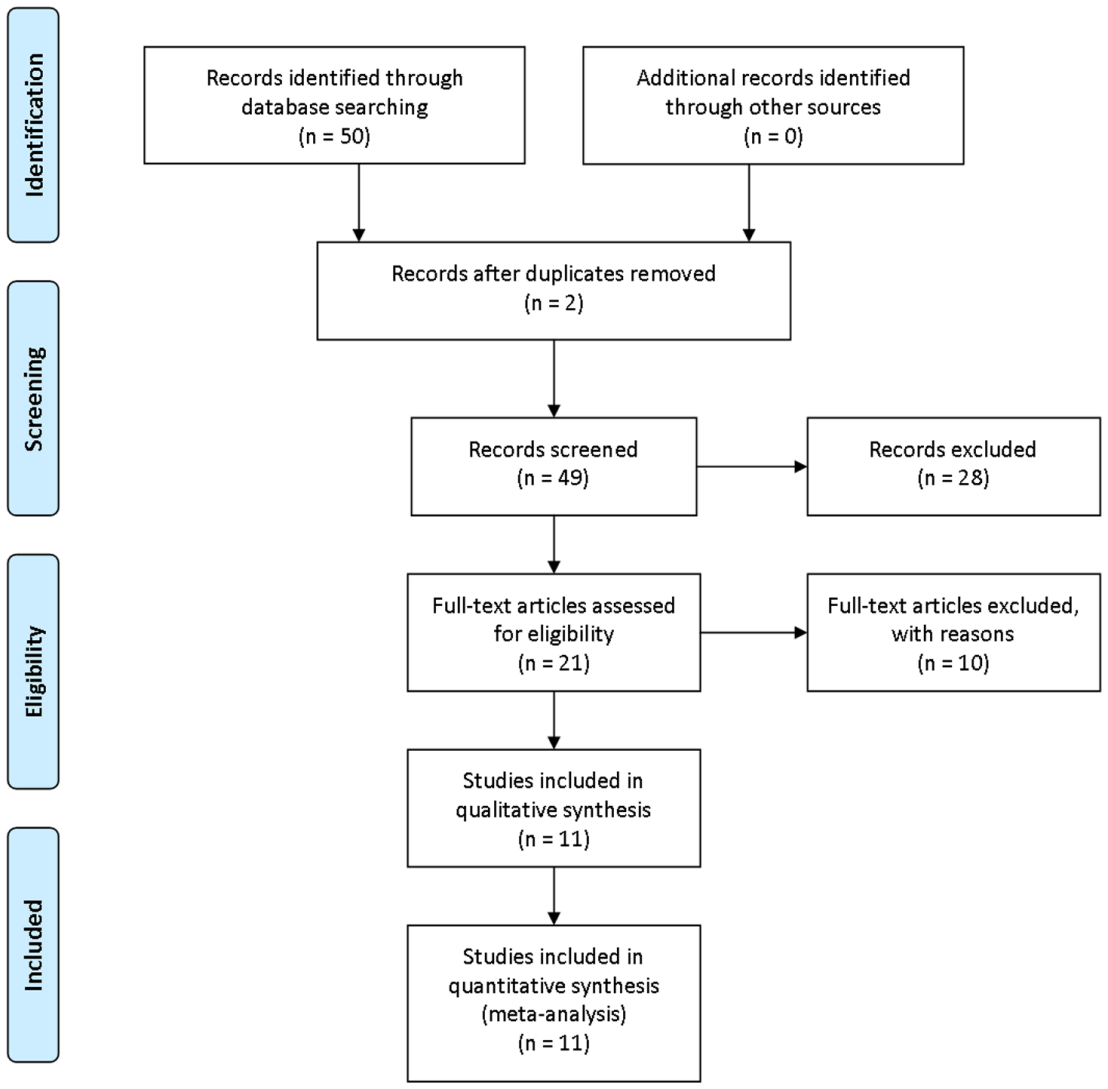
Flow diagram of study selection.

The electronic search identified 50 potentially relevant articles. One initial screening, 28 were excluded based on titles and abstracts, and they were studies with nonclinical studies, case reports, or lack of comparison group. Full-text evaluation were conducted in the remaining 21 articles, and 10 articles were excluded for not fulfilling our inclusion criteria: 3 articles reported insufficient data on serum UA levels; 3 articles did not apply well-accepted diagnosis standard for AD; 2 articles used CSF, not blood as specimen; 1 article was excluded due to no original data reported; 3 studies were based on the same population, the one containing the most complete and relevant data was used. Eventually, 11 studies were included in this analysis. The selecting process is listed in a flow diagram (Figure 1).

### Study characteristics

Eleven studies comparing the serum UA levels between AD patients and healthy controls, representing data from 2708 participants were included in the analysis. The characteristics of the studies and of their participants were shown in Table 1. All the eleven studies included both men and women. 1 studies reported gender-specific change of serum UA levels in their participants [43]. Of the 11 trials, 3 were conducted primarily in Italy [44], [45], [46], 1 was conducted in the United States [47] and 1 was performed in United Kingdom [48]. Three studies were done in Asian countries [35], [40], [49] and five studies were from European countries [44], [45], [46], [50], [51]. All the eleven studies applied the criteria “National Institute of Neurological and Communicative Disorders and Stroke-Alzheimer's Disease and Related Disorders Association” (NINCDS-ADRDA) for AD. Four studies used the criteria of the Diagnostic and Statistical Manual of Mental Disorder, 4th edition for dementia of the Alzheimer's type (DSM-IV) [35], [40], [48], [49]. Two study applied 3rd edition for dementia of the Alzheimer's type (DSM-III) [47], [51]. Mini-Mental State Examination (MMSE) was performed in 9 studies for cognitive assessment [35], [40], [44], [45], [47], [48], [49], [50], [51]. Clinical Dementia Rating Scale (CDR) was used in 2 studies to quantify the severity of dementia symptoms [40], [49]. Seven trials have additional experimental group (mild cognitive impairment group or vascular dementia group) plus control group [44], [45], [46], [47], [48], [50], [51]. The details of the characteristics of included studies were summarized in Table 1. The overall quality of studies was good, ranging from 6 to 9 (Table 2).

**Table 1:** Characteristics of included cohort studies.

Author, year, country	No. participants total/control	Gender, Age	UA, mg/dl	AD Definition	Variables controlled
Polidori, 2004, Germany	63/55	M/W 76.8	3.35±0.87	NINCDS-ADRDA criteria and neuroradiologic findings	age, sex, race, nutritional status, BMI, anxiety or depression, smoking, alcohol, antioxidant supplement
Carantoni, 2000, Italy	24/66	M/W 83	5±0.5	NINCDS-ADRDA criteria and neuroradiologic findings	age, sex, race, metabolic profiles, BMI, drug supplement
Cervellati, 2013, Italy	101/98	M/W 77.9	6.20±0.18	NINCDS-ADRDA criteria	age, sex, hypertension, cardiovascular disease, diabetes, and smoking
Cascalheira, 2009, Portugal	19/36	M/W 76	5.3±1.9	NINCDS-ADRDA criteria and neuroradiologic findings	age, sex, hypertension, cardiovascular disease, diabetes, smoking, alcohol, and drug supplement
Can, 2013, Turkey	32/32	M/W 72.4	4.51±1.03	DSM-IV, NINCDS-ADRDA criteria	age, sex, biochemical characteristics
Kim, 2006, Korea	101/101	M/W 73.5	4.4±1.2	DSM-IV, NINCDS-ADRDA criteria	age, sex, BMI, and MMSE
Cankurtaran, 2013, Turkey	143/1553	M/W 73.52	5.10±1.43	DSM-IV, NINCDS-ADRDA criteria and neuroradiologic findings	age, sex, nutritional status, biochemical characteristics, lipid profiles, hypertension, cardiovascular disease, diabetes, smoking, and drug supplement
Iuliano, 2010, Italy	37/24	M/W 76.03	4.95±1.38	NINCDS-ADRDA criteria and neuroradiologic findings	age, sex, primary psychiatric or neurological disorders, system diseases, smoking, alcohol, and drug supplement
Zafrilla, 2006, Spain	30/27	M/W 76	4.1±0.3	NINCDS-DSM-III criteria	age, sex, nutritional status, plasma antioxidants, lipid peroxidation profiles, system diseases, smoking, and drug supplement
Foy, 1999, UK	79/58	M/W 79	5.21±1.09	DSM-IV, NINCDS-ADRDA criteria and neuroradiologic findings	age, sex, nutritional status, plasma antioxidants, system diseases, neurological disorders
Maesaka, 1993, US	18/11	M/W 79.2	6.20±0.34	NINCDS-DSM-III criteria and neuroradiologic findings	age, sex, cognitive heart failure, malignant disease, liver disease

NINCDS-ADRDA = National Institute of Neurological and Communicative Disorders and Stroke-Alzheimer's Disease and Related Disorders Association, DSM-IV = the Diagnostic and Statistical Manual of Mental Disorder, 4th edition for dementia of the Alzheimer's type, DSM-III = 3rd edition for dementia of the Alzheimer's type, BMI = body mass index, M = men, W = woman, C = combined. Age range or median age.

**Table 2:** Quality assessment of included studies based on Newcastle-Ottawa Scale.

Author	Selection	Comparability	Exposure
Polidori	3	2	3
Carantoni	4	2	3
Cervellati	3	0	3
Cascalheira	3	2	3
Can	4	1	3
Kim	3	2	3
Cankurtaran	3	2	2
Iuliano	4	2	3
Zafrilla	3	2	2
Foy	4	2	3
Maesaka	3	2	3

### Risk of Bias in Included Studies

**Figure 2:**
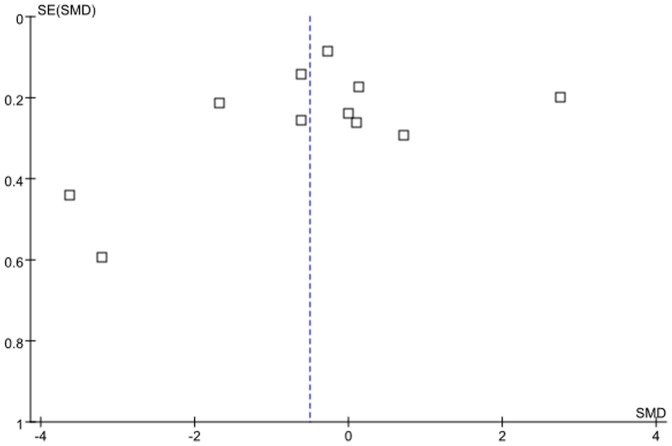
Publication bias from studies on assessment of serum UA levels in AD patients.

The funnel plot for the included 11 studies comprising AD patients and healthy controls was visually examined, and the shape of this lot was presented essentially symmetrical (Figure 2). There was no statistical evidence of a publication bia among studies either from the result of Egger's (p = 0.57) or Begg's (p = 0.42) tests.

### Sensitivity Analyses

In sensitivity analysis, we recalculated the combined standardized mean difference (SMD) by omitting one study per iteration. The study-specific combined SMDs ranged from a low of −0.78 (95% CI, −1.29,−0.27) via omission of the study by Cervellati et al, to a high of −0.55 (95% CI, −1.49,0.39) via omission of the study by Cankurtaran et al. When the study by Cervellati et al was included, the SMDs from the remaining studies yielded consistent negative results without great fluctuations, and the range of the combined SMDs was narrow. When this study was excluded, the 95% CI did not contain 0, a statistically significant difference was identified.

### Meta-regression

Since significant heterogeneity was found among individual studies, a meta-regression was performed to explore the predefined possible source of heterogeneity. None of the regression coefficients was statistically significant (Table 3), suggesting that year of publication, race, sample size, and mean age were no significant source of heterogeneity.

**Table 3:** Effect of study variables by meta-regression.

	Serum UA level	SMD
	Coefficient	95% CI
Year of publication	0.08	−0.12–0.28
Race	1.52	−1.42–4.46
Sample size	0.50	−2.59–3.60
Mean age	1.39	−3.93–6.71

### Serum UA levels in AD patients and healthy controls

**Figure 3:**
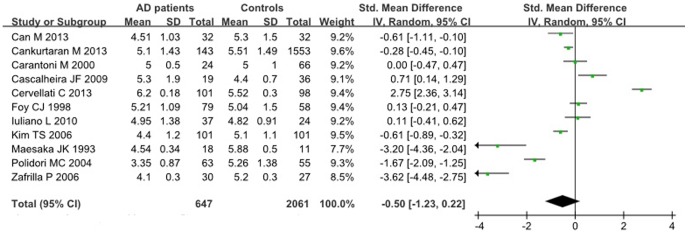
The serum UA levels in AD patients compared with healthy controls.

A significant heterogeneity across studies was found (I^2^ = 58%, p<0.01), thus the random-effects model was used to calculate the pooled effect size. Using all available data to compare serum UA levels among AD patients compared with controls, there were no significant differences between these two groups. The pooled SMD for the eleven studies on the random-effects model was (−0.50; 95% CI: −1.23, 0.22) (Figure 3).

## Discussion

In the meta-analysis derived from 11 case-control studies comparing serum UA levels between AD patients and healthy controls, we found that serum UA levels in AD patients did not differ significantly from that in healthy controls. However, some meta-analysis showed that serum UA levels were significantly lower in patients with multiple sclerosis (MS) [52], Serum UA levels were lower in PD patients than in controls [53], and higher serum UA levels were associated with lower PD incidence [54]. Many previous studies observed a trend toward decreased UA level in AD [35], [40], [44], [47], [49], [50], [51]. Based on these studies, it has been postulated that lower serum UA levels were associated with a higher risk of AD, since UA may play an antioxidant role. However, some recent studies found that serum UA levels were significantly higher in AD patients compared with controls [43], [45], [46]. In the present meta-analysis, serum UA levels in AD patients were not significantly different from those in healthy controls. This finding was consistent with the results from a present meta-analysis. They found that non-enzymatic antioxidants were significantly reduced in serum of AD patients, except for UA. They only identified a trend toward a decrease of serum UA levels in AD patients (p<0.1) [55]. There are several factors, which may contribute to the lack of a clear link between serum UA level and AD. First, the different methods to examine the serum UA level and varying test sensitivities among different studies. Also, most of the included studies had a small sample size. Thus, the statistical power may be insufficient to detect the difference of serum UA levels between AD patients and healthy controls. In addition, sensitivity is a major concern in meta-analysis. A possible low sensitivity was observed throughout our study. The result from the study conducted by Cervellati et al greatly influenced the overall results, thus the results of the meta-analysis couldn't be regarded with higher degree of certainty. When this study was excluded, significantly lower serum UA levels were identified in AD patients. We checked the original study from Cervellati et al, and found that controls were significantly younger than AD patients (mean age, 65.7 vs 77.9). Serum UA levels were strongly correlated with aging [56], and it steadily increased with advancing age in both men and women [57]. The age of AD patients in Cervellati's study may partly influence serum UA levels in AD patients. Another interference factor is hypertension. 66.5% AD patients in Cervellati's study had hypertension, while only 36.5% healthy controls carried hypertension, and the difference was significant. An independent positive correlation between serum UA levels and the occurrence of hypertension has known for some time [58]. Hypertension can cause local tissue ischemia, which can then lead to an increase in the synthesis of UA [59]. Furthermore, it is well established that that hypertension in the elderly is a potential risk factor to AD [60], [61], [62], [63]. It was hypothesized that chronic brain hypoperfusion generated by vessel stiffness and increased vascular resistance could be a key factor linking high blood pressure and AD [64], [65]. In addition, in Cervellati's study, compared to control group, the AD group had a higher proportion of patients with diabetes mellitus, cardiovascular disease (CVD), and history of smoking habit. It was reported that newly diagnosed diabetes mellitus was associated with increased risk of AD [66]. The presence of CVD was also reported to promote progressive cognitive decline leading to AD in the elderly population [67]. In particular, atherosclerosis, stiffening of central elastic arteries and endothelial dysfunction have been shown to associate with AD [68]. A variety of CAD-related risk factors, including hypertension, metabolic syndrome, smoking, high serum lipid/cholesterol levels, high serum homocysteine, physical inactivity, and unhealthy diet have been reported to play a role in the etiology of AD [69]. Serum UA levels were found to be significantly higher in patients with CAD compared with healthy controls [70]. Increased UA levels were also considered to be an independent risk factor for overall CAD mortality [71]. Thus, hyperuricemia may also be a risk factor for CAD [72], [73]. The positive association between serum UA levels and the risk of CAD may be due to the role of UA as an antioxidant [31], [74]. Increased UA levels, with an attempt to block lipid peroxidation and other related phenomena, could be a defense mechanism against atherosclerosis. On the other hand, increased UA levels may contribute to the development of CAD by exerting a negative influence on the endothelium. It was reported that serum UA could possibly promote oxygenation of low-density lipoprotein cholesterol and lipid peroxidation, lead to an increase in platelet adhesiveness and thrombus formation, and contribute to the development of atherosclerosis [75]. Increased UA levels can also stimulate the release of free radicals, and result in endothelial injury, increasing the likelihood of the development of CAD [76]. Elevated UA levels, as CAD-related risk factors, could play a role in the pathogenesis of AD in Cervellati's study. Thus, the higher presence the risk factors, including aging, hypertension, diabetes mellitus and smoking in AD group tends to increase the probability of acquiring AD, and these factors may be related to the increased serum UA levels in AD patients. Considering these factors, we recommend that the overall results must be interpreted with an appropriate degree of caution.

We also want to emphasize that vascular disease and its risk factors, mainly affecting the elderly population, have become an important risk factor in the development of cognitive decline and AD. These factors might also play a vital role in the initiation of dementia in AD by possibly sharing common pathological pathways. Chronic brain hypoperfusion leads to a neuronal energy crisis, which may be responsible for protein synthesis abnormalities, resulting in the formation of amyloid-beta plaques and neurofibrillary tangles in AD [67]. In this way, AD could be considered as a primary vascular disorder [77]. Moreover, the therapeutic implications of identifying and treating all modifiable risk factors may thus help lower the rising prevalence of AD [78]. In fact, the manipulation of serum UA levels has been investigated for the treatment of several diseases. Human studies have shown that systemic administration of UA increased the serum antioxidant capacity in healthy volunteers [79]. In various models of ischemic brain injury, UA could reduce ischemia-induced tyrosine nitration and infarct volume. In patients with acute stroke, administration of UA could reduce several biomarkers of oxidative stress and provide neuroprotection synergistically with thrombolytic therapies [80], [81]. In a mouse model of MS, UA could delay the onset and improve the clinical symptoms of disease by inhibiting peroxynitrite-mediated oxidation [82], [83]. UA could protect cultured rat spinal cord neurons against glutamate toxicity, and UA treatment could increase total glutathione (GSH) synthesis both in vitro and in vivo to fight against oxidant insult [79], [84], [85]. In animal models and in vitro cell models of PD, UA could suppress oxidative stress, inhibit dopamine-induced apoptotic cell death, and prevent membrane depolarization and cell degeneration [86]. In a 6-OHDA rat model of PD, UA injection significantly improved dopamine depletion and related behavioral responses [87]. In PD patients, phase II clinical trial known as SURE-PD (Safety and Ability to Elevate Urate in Early Parkinson Disease) is in progress at 16 clinical sites, which will test inosine as UA precursor to modify PD progression [86].

The pathogenetic role of UA in AD was still unknown. UA, the main end product of purine metabolism, is synthesized by xanthine oxidoreductase from its purine precusors [76]. Primarily through its actions as a potent antioxidant, UA scavenges singlet oxygen, hydroxyl radicals, and superoxide. UA is also capable to bind iron and inhibit iron-dependent ascrobate oxidation, preventing the generation of free radicals [74]. UA is effective in inhibiting peroxynitrite through nitrating the tyrosine residues of proteins [88]. Peroxynitrite, in particular, is believed to have a significant negative impact on cellular function and survival [89]. Thus, reduced serum UA level may weaken the body's ability to inhibit the toxicity exerting by reactive oxygen, nitrogen species, and peroxynitrite.

The limitations of this meta-analysis fall into four categories: First, as our results were based on observational studies, and we must be aware of potential bias or confounding in these studies. Second, we were unable to stratify these individual studies by the gender due to the lack of sex-specific study data. Previous studies suggested the gender-related association between serum UA levels and hypertension [90], type 2 diabetes [91], CAD [92], PD [93], MS [94], and amyotrophic lateral sclerosis (ALS) [95]. Estrogen has an impact on the renal tubular handling of UA [96]. Study has found that menopause was associated with higher serum UA levels, and that postmenopausal hormone replacement was linked with lower serum UA levels among postmenopausal women [97]. These findings suggested that estrogen plays a key role in protecting women from hyperuricemia and gout [97]. In addition, administration of estrogen could decrease serum UA levels in men [98]. One possible explanation for the gender difference of UA in a variety of diseases could be caused by different estrogen levels in men and women. Therefore, whether the change of serum UA levels in AD patients is affected by gender difference still needs further more sex-specific studies. Third, several residual confounding variables may affect the overall results. For example, high intake of purine-rich food [99] and fructose [100] may promote the development of hyperuricemia, and the use of medications such as diuretics was not considered in some included studies. Thus, further studies related to diet, nutrition profile, systemic diseases, and drug-taking history will be needed to validate the relationship of serum UA to AD. Finally, regarding the strict inclusion criteria which only include case-control studies, our study contains a limited number of studies. Because meta-analyses are inherently vulnerable to publication bias, we cannot exclude the possibility of this bias. We try to minimize publication bias by searching four major databases with no language restriction; Egger's and Begg's tests reveal no statistical evidence for significant publication bias.

Our meta-analysis has several important strengths. This meta-analysis is based on case-control studies in many different countries. To our knowledge, this is the first systematic review and meta-analysis to compare the serum UA levels between AD patients and healthy controls. We assessed the quality of each study using the Newcastle–Ottawa Scale, and all of them were high quality. The majority of the included studies had adjustments for other risk factors. However, heterogeneity between studies was found, and it was affected by one study in the sensitivity analysis. Although studies differed by publication of year, race, sample size, and mean age, meta-regression analysis did not find any significant association with these factors. Nonetheless, the observed heterogeneity could be attributable to differences in countries, environmental factors, methodological factors in design, and clinical features. As mentioned before, the presence of heterogeneity calls for caution in interpreting the present meta-analysis findings.

## Conclusion

Our meta-analysis of case-control studies suggests that serum UA levels do not differ significantly between AD patients and healthy controls, but there may be a trend toward decreased UA in AD after an appropriate interpretation. Future research should be well-designed to investigate serum UA concentrations in AD patients, and more attention should be paid on confirming the pathogenetic mechanisms of UA as well as examining the role of UA-manipulating therapy in AD.

## Supporting Information

Checklist S1 PRISMA Checklist for systematic review and meta-analysis.
(DOC)
